# In Vitro Assessment of a New Block Design for Implant Crowns with Functional Gradient Fabricated with Resin Composite and Zirconia Insert †

**DOI:** 10.3390/ma17153815

**Published:** 2024-08-02

**Authors:** Nicolás Gutiérrez Robledo, Miquel Punset Fuste, Alejandra Rodríguez-Contreras, Fernando García Marro, José María Manero Planella, Oscar Figueras-Álvarez, Miguel Roig Cayón

**Affiliations:** 1 School of Dentistry, Universitat Internacional de Catalunya (UIC), 08195 Barcelona, Spain; ofigueras@uic.es (O.F.-Á.); mroig@uic.es (M.R.C.); 2 Independent Research, 28028 Madrid, Spain; 3 Biomaterials, Biomechanics and Tissue Engineering Group (BBT), Universitat Politècnica de Catalunya (UPC), 08019 Barcelona, Spain; miquel.punset@upc.edu (M.P.F.); alejandra.maria.rodriguez@upc.edu (A.R.-C.); jose.maria.manero@upc.edu (J.M.M.P.); 4 UPC Innovation and Technology Center (CIT-UPC), Universitat Politècnica de Catalunya (UPC), 08034 Barcelona, Spain; 5 Barcelona Research Centre in Multiscale Science and Engineering, Universitat Politècnica de Catalunya (UPC), 08019 Barcelona, Spain; fernando.garcia.marro@upc.edu; 6 Reserach Institute San Joan de Déu (IRSJD), 08950 Barcelona, Spain; 7 Center for Structural Integrity, Reliability and Micromechanics of Materials Research Group (CIEFMA), Universitat Politècnica de Catalunya (UPC), 08019 Barcelona, Spain

**Keywords:** ceramic-reinforced resin-composite blocks, monolithic dental crown, layered dental crown, zirconia insert, dental fatigue

## Abstract

This study aims to evaluate and compare the mechanical resistance, fatigue behavior and fracture behavior of different CAD/CAM materials for implant crowns. Eighty-eight implant crowns cemented-screwed with four sample groups: two monolithic G1 Zirconia (control) and G3 composite and two bi-layered G2 customized zirconia/composite and G4 prefabricated zirconia/composite. All static and dynamic mechanical tests were conducted at 37 °C under wet conditions. The fractographic evaluation of deformed and/or fractured samples was evaluated via electron microscopy. Statistical analysis was conducted using Wallis tests, which were performed depending on the variables, with a confidence interval of 95%, (*p* < 0.05). The Maximum Fracture Strength values displayed by the four groups of samples showed no statistically significant differences. The crown–abutment material combination influenced the failure mode of the restoration, transitioning from a fatigue fracture type located at the abutment–analog connection for monolithic materials (G1 and G3) to a brittle fracture located in the crown for bi-layered materials (G2 and G4). The use of layered crown materials with functional gradients appears to protect the crown/abutment connection area by partially absorbing the applied mechanical loads. This prevents catastrophic mechanical failures, avoiding long chairside time to solve these kinds of complications.

## 1. Introduction

The absence of the periodontal ligament in the implant–prosthesis–bone set can frequently generate mechanical complications in implant prostheses. This occurs due to the mechanical overload caused by the lack of cushioning [1], especially in the unitary crowns of male molars [2,3]. The most frequent mechanical complications are the loosening of the fixation screw or the transepithelial abutment [4,5] and the fracture or delamination (chipping) of the esthetic coating material of the restoration [6].

The mechanical properties of the ceramic-reinforced resin composite with a dispersed filler microstructure (DFM) included in the new classification of ceramic materials [7], have considerably improved due to new industrial polymerization methods (atmospheric pressure and high temperature) [8,9], which replace intraoral photopolymerization [10]. A reduced monomer release and an increase in flexural strength, hardness, tenacity and even density of these materials have been observed [11,12,13,14]. The great stability achieved in resin-composite blocks (RCBs) allows excellent reproducibility by machining [15,16] and causes less wear on the opposing enamel compared to glass-matrix ceramics [17,18,19,20]. All these characteristics, together with the low flexural modulus, allow RCBs to achieve a cushioning effect on implant restorations by transmitting less stress to the prosthetic attachment and implant, which favors bone response [21,22,23,24,25]. On the other hand, when the implant–prosthesis–bone complex exceeds the limits of physiological adaptation, it produces bone overload, which, in turn, triggers osteoclastic activity and bone resorption [26,27,28]. This sometimes results in traumatic failure, diverging from infectious failure or bacterial peri-implantitis [29].

Marginal bone preservation is influenced by multiple factors, such as implant design, crown material, prosthetic abutment, connection and disconnection of the prosthetic abutment and the type of surgery performed. The correct selection of the restorative material, as well as the prosthetic abutment, is one of the keys to success in implant prosthesis [30,31,32]. Given that the masticatory force into the crown is then transmitted to the maxillary or mandibular bone through the prosthesis and implant [33], the use of resilient materials that function as a shock absorber to the implant–prosthesis set could help reduce stress and pressure on the bone [34]. It may reduce prosthetic complications derived from the overload received in the crown [35]. Currently, the prefabricated titanium abutment with a mesostructured zirconia to mask the grayish color of titanium has become the prosthetic abutment of choice for restorations on implants, given its excellent mechanical and esthetic properties as well as its biocompatibility with the tissues [36,37,38].

Functional gradient materials (FGMs) are a new concept in materials engineering [39], where both the material composition and structure gradually change. Their sole purpose is to dissipate or absorb stress or mechanical load.

FGMs have been used in several investigations in the dental field [40,41,42]. In this process, both the material composition and the structure gradually change throughout the volume, resulting in changes in the material’s properties, chemical composition, physical state and geometric configuration. This has allowed us to explore optimal designs of bio-inspired or bio-mimetic materials in which different layers of materials achieve greater stress reduction and distribution. The best example is the human tooth, which consists of two main layers: enamel and dentin. The outer layer allows it to maintain its shape and resist fracture and wear upon loading. This quality is due to a variation of the transformation in its microstructure and chemical composition. Starting from the outermost layer, four well-differentiated histological layers are recognized until reaching the dentin–enamel junction (DEJ) [43,44]. This interface plays a crucial role in the fatigue resistance of the natural tooth, given the reliable connection between the different layers of the tooth [45,46]. This study is the first one in the field and is unique because it combines different materials perfectly bonded to produce a functional gradient implant crown, trying to emulate the natural tooth structure.

**Figure 1:**
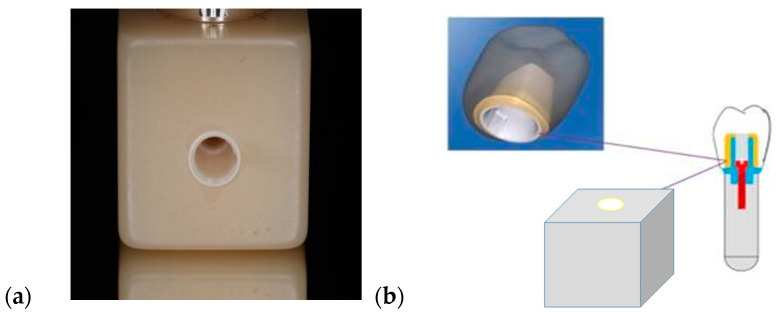
Experimental setup: (**a**) resin composite experimental block with prefabricated zirconia insert and (**b**) implant crown cemented over Ti-base and screwed on the implant analog.

The reproducibility of the resin composite by machining and the feasibility of polishing or intraoral adjustment allow great control of the occlusion. Moreover, the favorable cutting properties lead to considerable savings in production time and reduce wear on manufacturing instruments, such as motors and diamond milling cutters; in contrast, materials with a ceramic matrix, when machined, often result in lower-quality margins and edges [16]. A layered design with an individualized zirconia mesostructure implies additional production costs, as the mesostructure must be designed and milled separately from an additional block and then requires an additional cementation to the resin composite crown. As a solution, a new resin-composite block design with an industrially bonded zirconia insert is proposed (Figure 1). The industrially manufactured insert, which is a component of the resin block, enables the masking of the metal’s colors and ensures an optimal fit tolerance to the titanium base. In addition, it can be used for chair-side restorations on implants, as it can be machined within 12–14 min and does not require thermal processing.

The design of the new experimental block is composed of two materials commonly used today: resin composite with filler content of VOL % 51.5 and zirconia. These two materials are industrially bonded to ensure optimal and consistent tolerances. From a mechanical and an esthetic point of view, the experimental crown could be an effective prosthetic solution with the combination of prefabricated zirconia insert and resin-composite block [35,47]. Furthermore, it is easy to polish and repair in the mouth. Additionally, due to its low production cost, it could be affordable for most patients.

This study aims to evaluate the mechanical resistance of the resin-composite blocks for implant crowns under different scenarios. We will compare fracture behavior and failure mode with the control group monolithic zirconia, one of the most common materials for single-unit implant crowns under static and dynamic loads.

The study’s main hypothesis suggests the presence of statistically significant differences in both maximum static strength and fatigue limit among the materials evaluated. However, the null hypothesis in this study proposes the absence of statistically significant disparities in these properties, regardless of the material’s characteristics and the component’s geometry.

## 2. Materials and Methods

### 2.1. Materials

**Figure 2:**
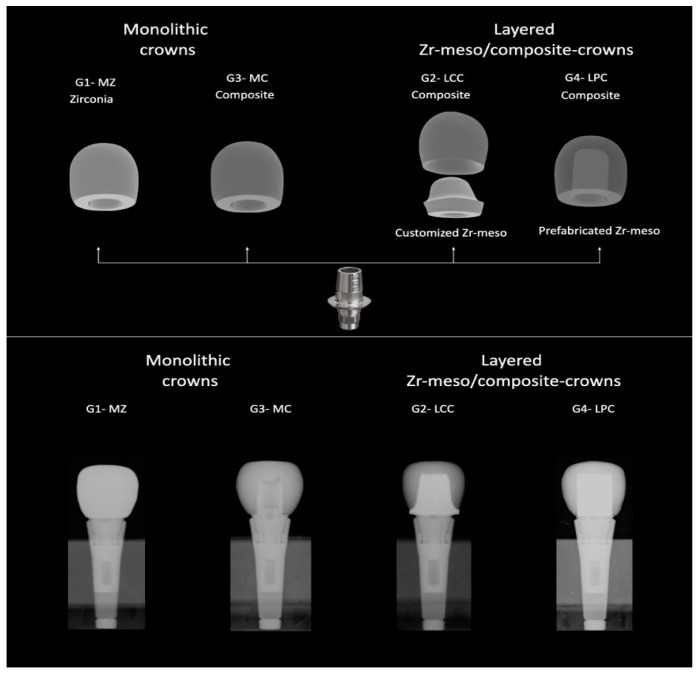
Different study groups and X-rays of samples tested: monolithic zirconia (G1-MZ), monolithic composite (G3-MC), layered customized composite (G2-LCC) and layered prefabricated composite (G4 LPC).

Eighty-eight ASTRA EV implant analogs (Dentsply Sirona, Charlotte, NC, USA) with a diameter of XL 5.4 mm (Ref 25547 and LOT 456009) were prepared with the corresponding CEREC Ti-base (CEREC/inLab Ref 6586338 and LOT B200003054), onto which a crown of each group (n = 22) was previously cemented. The sample groups were designated as G1 (control group) and G3, which comprised monolithic crowns, whereas the sample groups G2 and G4 comprised layered crowns with customized or prefabricated mesostructured zirconia (Figure 2).

### 2.2. Sample Preparation

All samples were prepared and treated according to ISO 14801:2016 [48]. The preparation of the samples to be mechanically tested in this study was carried out in two consecutive and well-differentiated sequential stages. The first stage involved embedding the ASTRA EV implant analogs in a bone-like resin, followed by the second stage of fabricating and assembling the different crowns by cementation.

Before performing any static and dynamic mechanical testing, all titanium analogs were inserted into a bone-like polymeric resin to provide stable support, as well as to mimic oral conditions. Analog samples were embedded into a polymeric resin (Mecaprex MA2+, PRESI SAS, Eybens, France), leaving the implant 3 ± 0.1 mm above the implant nominal bone level determined by the implant manufacturer. All samples were embedded, resulting in a total set of 88 samples for the study. The resin discs with the embedded implants were subjected to rectifying operations, thus ensuring parallelism between the upper and lower faces.

The second phase of sample preparation included the fabrication of four distinct crown groups, along with their final embedment into the analog, which had been previously fixed in a resin-like bone material during the preceding stage. The Ti-base was scanned with a Dentsply Sirona Ineos X5 extraoral scanner (Dentsply Sirona, Charlotte, NC, USA) using the corresponding “L” scan-body for the Ti-base. Once scanned, a crown was designed using the InLab CAD 19 software (Dentsply Sirona, Charlotte, NC, USA). An STL model of the monolithic crown groups G1-MZ and G3-MC and the layered crown groups G2-LCC and G4-LPC was obtained. The material’s characteristics are described in Table 1.

The single blocks for crowns of the G1-MZ, G2-LCC and G4-LPC groups were milled using the MCXL, a 4-axis milling machine (Dentsply Sirona, Charlotte, NC, USA), serial no. 106352, using the wet strategy and diamond burs. For the crowns of G3-MC, a 5-axis Imes icore 350 PRO milling machine (Imes Icore GmbH, Eiterfeld, Germany) was used. In addition, the Brilliant Crios Disc (Coltene Whaledent, Altstätten, Switzerland) was used and a CAM Mill Box software v5 SMART(CIM Systems s.r.l., Milan, Italy). Then, all the crowns were carefully separated from the blocks and discs using a cutting disk. Once the supports were polished, the zirconia crowns from G1 and the customized mesostructured from G2 were dried in a pre-drying oven (Imes Icore) for 15 min to remove the moisture from the wet milling. Subsequently, they were sintered in the corresponding program of the DEKEMA sintering furnace model AUSTROMAT 674 (DEKEMA Dental-Keramiköfen GmbH, Freilassing, Germany).

Before cementing all the crowns, the Ti-bases were attached to the implant analogs according to the manufacturer recommendations using a torque wrench with 25 Ncm, and then they were cemented over a Ti-base and finally, the crown holes were filled with Teflon tape and light-cure composite Brilliant EverGlow A2/B2 and One coat 7 Universal (Coltene Whaledent, Altstätten, Switzerland) as bonding. Lastly, the composite fillings were polished using silicon polishers DIATECH Shape guard (Coltene Whaledent, Altstätten, Switzerland). The surface treatment during the cementation of the different crown samples is described in Table 2.

### 2.3. Observation by Field Emission Scanning Electron Microscope

Scanning Electron Microscopy (SEM) enables the surface-level and comprehensive evaluation of components and samples by acquiring high-resolution images using the interactions generated between an incident electron beam and the surface under analysis. A Field Emission Scanning Electron Microscope (FSEM) model JSM-7001F Scanning Microscope (JSM 7100, JEOL Ltd., Akishima, Japan) was used for fractographic evaluation of deformed and/or fractured specimens, operating at a potential of 20 kV and an approximate working distance ranging from 9 to 11 mm. This equipment is equipped with an Energy-Dispersive X-ray Spectroscopy (EDS) analysis probe, Oxford Xmax20 model, which allows for the identification of chemical composition by acquiring the characteristic X-ray emission of each chemical element.

Coating ceramic samples for SEM observation is essential to improve conductivity, prevent charge accumulation, protect the sample, and obtain higher quality and resolution images, enabling a more precise and detailed analysis of the properties and characteristics of this type of material. Once the samples had been fractured through fatigue testing, the fragments were positioned on pin-shaped holders to undergo a coating process using PVD-Sputtering techniques, specifically the LEICA EM ACE600 equipment (LEICA MICROSYSTEMS, Wetzlar, Germany). Using this equipment, a PVD-Sputtered Pt-Au conductive coating was applied to the samples prior to the SEM observation; this coating had an average thickness ranging from 5 to 10 nm.

### 2.4. Determination of the Maximum Compression Strength

**Figure 3:**
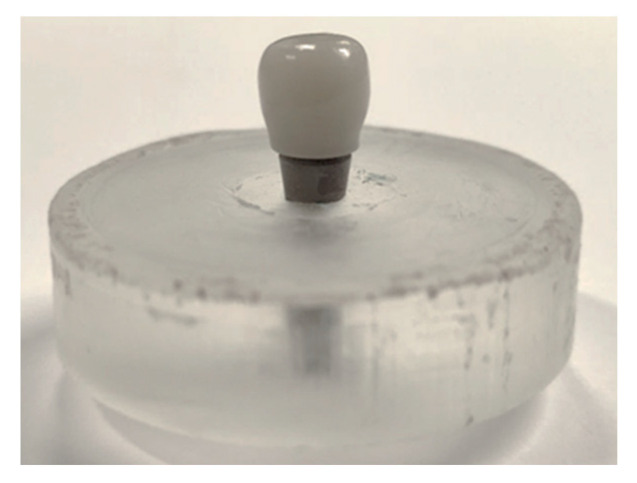
Image of the dental implant system embedded in the resin ready for testing.

A universal MTS model BIONIX-370 servo-hydraulic mechanical testing machine (MTS Bionix 370, Minneapolis, MN, USA) was used for the determination of the maximum compressive strength using a 2.5 kN load cell controlled by Telstar II software (Telstar, MTS System Corp., Eden Prairie, MN, USA). A total of 20 uniaxial static compression tests were carried out, divided into 5 tests for each of the 4 study sample groups to be evaluated. All static and dynamic mechanical tests were conducted at (37 ± 1) °C, fixing the specimen in the testing machine with a 30° angle of inclination and under wet conditions using Hank’s salt solution as a liquid medium. All the analyses were carried out under the same test conditions. The implants were held with the same and unique clamping device, consisting of a clamping jaw made of stainless steel, which supports the resin block in which each implant has previously been encasted (Figure 3). The compressive load was applied at a constant displacement rate of 1 mm/min on the loading device (cap) until system failure.

**Figure 4:**
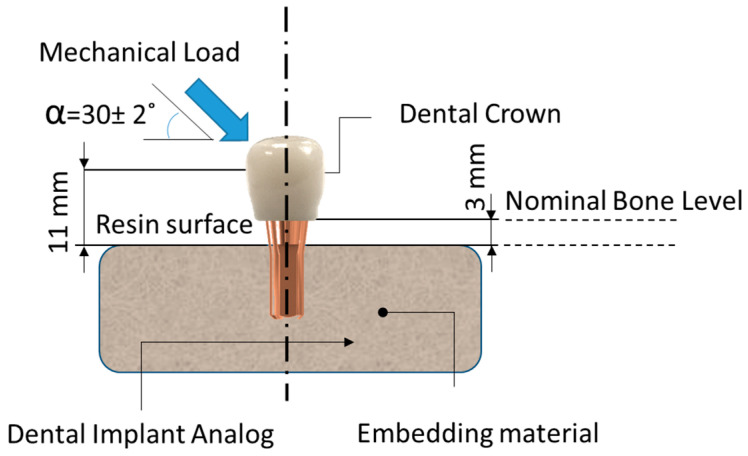
Schematic diagram of an embedded sample.

All samples were prepared following ISO-14801:2017. According to the standard, the bone anchoring part of the sample must be fixed in a fixed anchoring device that must hold the sample at a distance of 3.0 ± 0.1 mm apically from the nominal bone level determined by the manufacturer (Figure 4); in this case, the company Astra implants by Detsply Sirona. This distance is internationally accepted as the average level of bone resorption after dental implant implantation. The ISO-14801:2017 standard also specifies the existence of a constant distance of 11.0 ± 0.1 mm from the implant support level to the center of the hemispherical free end. This distance must be measured parallel to the central longitudinal axis of the implant body, and it is counted from the surface of the resin to the center of the hemispherical dome.

### 2.5. Determination of S-N Curve

Dental implant fracture is a critical concern in prosthetic dentistry. Cyclic loads experienced during mastication can potentially lead to structural failures, compromising the longevity and functionality of dental implants. Hence, it is crucial to evaluate the resistance of implants under varying cyclic load conditions to ensure their reliability and durability. The ISO14801 standard provides guidelines for testing the fatigue strength of dental implants, offering a standardized approach for assessing their performance. After conducting static compression-to-fracture tests, fatigue tests were carried out at various percentages of the previously obtained maximum breaking load. This allowed determining the number of cycles before fracture at each load level (n ≤ 4), starting from an initial load of 80% according to ISO 14801 of the load to failure in a static test carried out with the same test geometry. Following this guideline, the implants were submitted to a sinusoidal compression–compression fatigue test at a frequency of 2 Hz and a stress variation of 10%.

The total number of cycles applied to each sample was fixed at 2 × 10^6^, also defined as run-outs for fatigue tests performed in liquid immersion according to the ISO standard. Implants that survived this number of cycles were considered to have passed the test successfully. The force of the impact was performed on the distal cuspid of the implant-supported restoration for all groups of samples. Implants that endured this number of cycles were considered to have passed the test successfully. The fatigue test was run in liquid immersion using Hank’s salt solution (Sigma Aldrich, St. Louis, MO, USA) as a liquid medium. 

### 2.6. Characterization of Hardness and Fracture Toughness

The determination of hardness was analyzed by using a Vickers EMCO-Test microhardness tester (EMCO-TEST Prüfmaschinen Gmbh, Kuchl, Austria) equipped with a Vickers indenter, which consists of a diamond pyramid with a base angle of 136°. The standard used for the determination of the hardness of the materials under study was ASTM E384-17 [49].

The hardness measurements were carried out under a constant load of 5 kg applied for 15 s, making a total of three measurements for each of the four materials studied. The Vickers hardness number (VHN) (GPa) was obtained and compared. The Vickers hardness number (VHN) in GPa has been expressed following Equation (1). VHN as a function of the applied load (F) in N and the average of the diagonals of the indentation (d) in µm. The constant value, 1854.4, was obtained from the calculation of the contact area.

(1) $$VHN1854.4\times \frac{P}{d^{2}}$$

**Figure 5:**
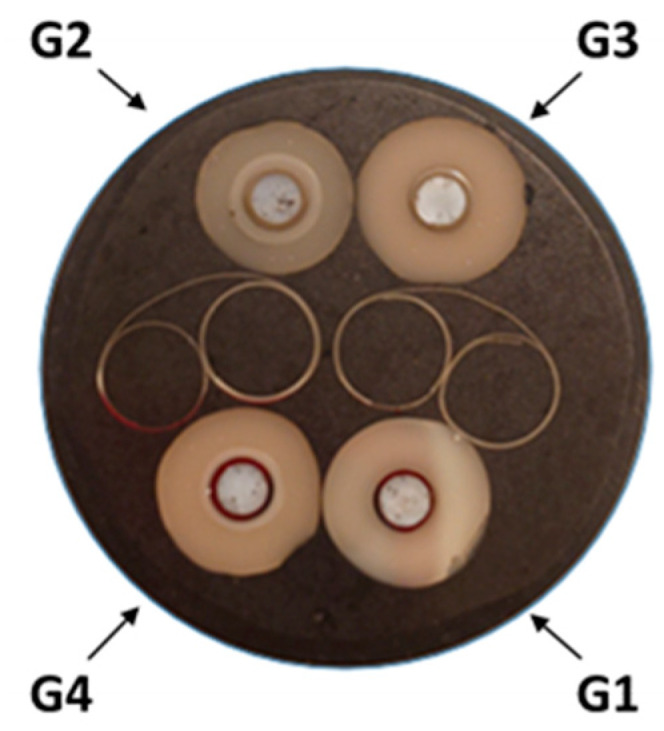
Picture of the polished cross-sections of G1, G2, G3 and G4 material.

The estimation of fracture toughness $K_{Ic\;}$ [49,50,51,52,53,54] was achieved through the measurement of crack length nucleated at the corners of the residual imprint, utilizing indentation fracture toughness, following research by Niihara et al. [55]. Sample preparation was necessary to produce a polished cross-section for each pillar (Figure 5).

The fracture toughness $K_{Ic}$ [50] is a crucial mechanical parameter in brittle materials that quantifies their ability to resist crack propagation. The estimation of fracture toughness can be achieved through the measurement of crack length nucleated at the corners of the residual imprint, utilizing a technique known as indentation microfracture. Various mathematical formulations have been suggested to determine $K_{Ic}$, contingent upon the tip indenter geometry and crack morphology (such as radial, half a penny, or Palmqvist). Among these, the expression most commonly employed for radial cracks is Equation (2) [51]:

(2) KIC=aEH12×PC32

where *a* is a (dimensionless) empirical constant depending on the indenter geometry (*a* = 0016 for pyramidal tips), *P* (in mN) is the peak indentation load, and *C* (in mm) is the length of the radial cracks. For Palmqvist cracks, the following Equation (3) applies [52]:

(3) KIC=al12×EH23×PC32

where xv is 0.016 for a Berkovich tip indenter, *a* (mm) is the length from the center of the imprint until one of the corners, and *l* (mm) is the crack length. The applicability of the different expressions for indentation microfracture tests performed with Berkovich indenters has been extensively discussed in [53,54].

### 2.7. Statistical Analysis

Statistical analysis has been carried out using the statistical software Minitab^®^ 16.2.1 (Minitab Inc., State College, PA, USA). Parametric ANOVA or a non-parametric test with Kruskal–Wallis was performed, depending on the variables, with a confidence interval of 95% and considered statistically different when *p* < 0.05. Maximum compression strength results are set out as mean ± standard deviation. 

All data were analyzed, beginning with a normal distribution test to determine if the data followed a normal distribution. If the values followed a normal distribution (*p* > 0.05) and two independent data groups were compared, the statistical study was conducted using the parametric t-test. If the values followed a normal distribution (*p* > 0.05) and three or more independent data groups were compared, the statistical study was conducted using the ANOVA test. In both studies, the initial hypothesis assumed that all means were equal. To accept this initial hypothesis as true, the probability was set within a 95% confidence interval, meaning the probability of it not being true was only 5%. Therefore, when the probability is less than 0.05, it indicates that the hypothesis is not met and that the means are not equal. Thus, if *p* < 0.05, the means are different, indicating statistically significant differences.

If the values did not follow a normal distribution (*p* < 0.05) and two independent data groups were compared, the study was conducted using the non-parametric Mann–Whitney test. If the values did not follow a normal distribution (*p* < 0.05) and three or more independent data groups were compared, the study was conducted using the non-parametric Kruskal–Wallis test. Therefore, when the Mann–Whitney and Kruskal–Wallis probability is *p* < 0.05, there are statistically significant differences between the variables and the factors analyzed.

## 3. Results

### 3.1. Uniaxial Flex-Compression Resistance

**Figure 6:**
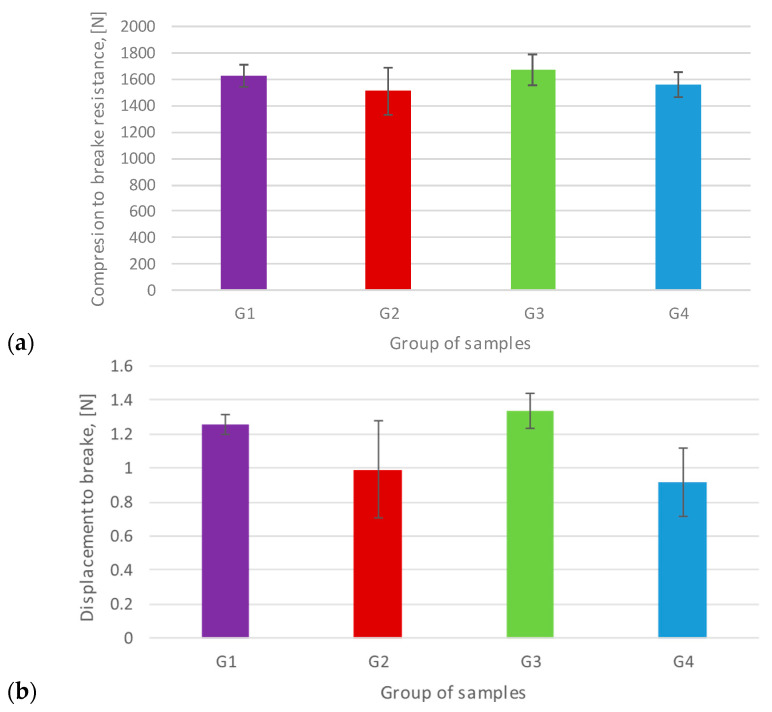
Comparative graphs of Fmax (**a**) and displacement to break (**b**).

To obtain average values of the static compression strength for all tested implant sample groups, it is necessary to determine the starting point for the different levels of load required to create an S/N curve. In order to do this, compression tests were conducted on five different samples (n = 5) per sample group (Table 3). The comparative analysis of the maximum static fracture strength values suggested slightly higher maximum strength values in groups G3 and G1 in comparison to groups G4 and G2, respectively (Figure 6a). However, the statistical analysis of maximum fracture force results of the four sample groups did not reveal the presence of statistically significant differences (*p* = 0.213).

**Figure 7:**
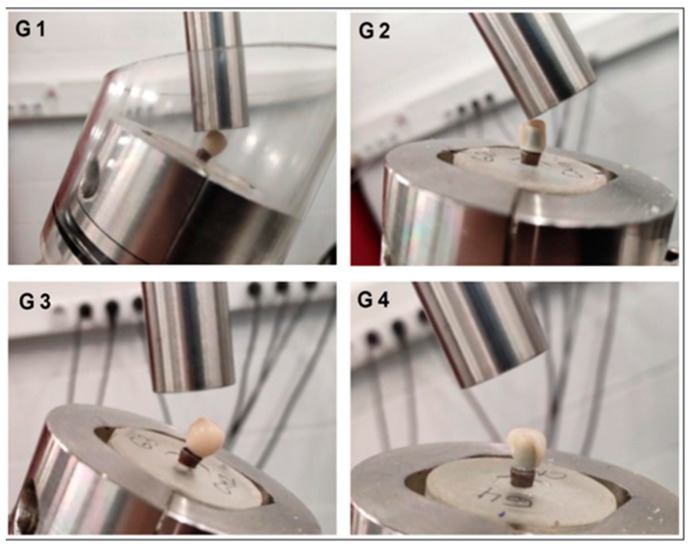
Photographic images of the fracture modes under static uniaxial compression.

Moreover, the comparative analysis of displacement at break proposed higher displacement values in groups G3 and G1 compared to groups G2 and G4, respectively (Figure 6b). Furthermore, the analysis conducted on the displacement-to-fracture outcomes within the four groups unveiled the existence of statistically significant disparities in displacement (*p* = 0.005). There were statistically significant differences from displacement-to-fracture between the G3 and G4 sample groups (*p* = 0.006) but not between G1 and G2 (*p* = 0.71). The four study groups showed different modes of fracture. They were characterized by a deformation of the neck of the analog and a fracture of the fixation screw in groups G1 and G3, and crown fracture in groups G2 and G4 (Figure 7).

The images in Figure 7 show the different modes of fracture exhibited by the analyzed samples of the four study groups. They are characterized by a crown fracture in groups G2 and G4, a deformation of the neck of the analog and the fracture of the fixation screw in groups G1 and G3.

### 3.2. Uniaxial Cyclic Fatigue Test

**Figure 8:**
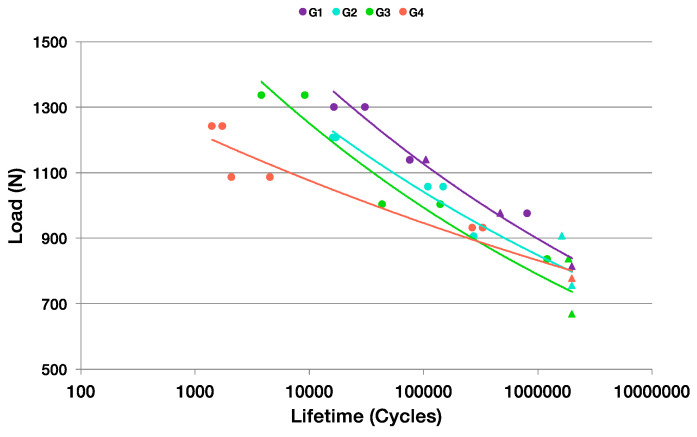
Comparative graph of S-N curves obtained for all groups of samples tested.

The S-N graphics obtained from the tests showed similar decreasing tendencies for all the groups (Figure 8). Moreover, the fatigue limit obtained was very similar for groups G2 and G4, whereas the G1 group showed the highest fatigue limit. Table 4 summarizes the fatigue limit (FL) for all sample groups.

**Figure 9:**
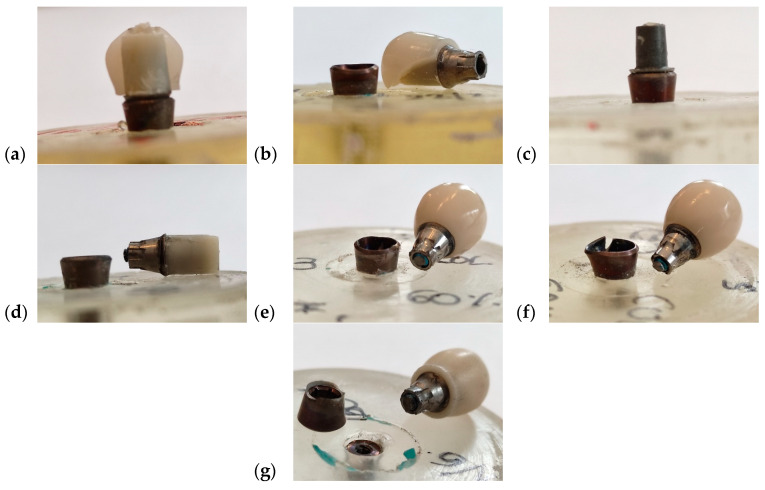
Photographic images of the different fracture modes identified: (**a**) T1, (**b**) T2, (**c**) T3, (**d**) T4, (**e**) T5, (**f**) T6 and (**g**) T7.

Moreover, Table 5 indicates the loads supported by each group sample and shows up to eight different fracture modes, from T1 through T8, with the eighth being run out of the samples. Likewise, Figure 9 is the illustrative image of these fracture modes on the samples.

A comparative analysis of the failure modes observed in this study has also revealed discrepancies among the different groups of samples evaluated. The implants in groups G2 and G3 exhibited very similar fracture behaviors, characterized by the same types of fracture modes at equivalent percentages of applied cyclic load. However, the G1 and G4 groups of samples not only displayed variations in fracture modes between each other but also demonstrated distinct fracture behaviors compared to the remaining groups, along with a greater variability of fracture modes.

**Figure 10:**
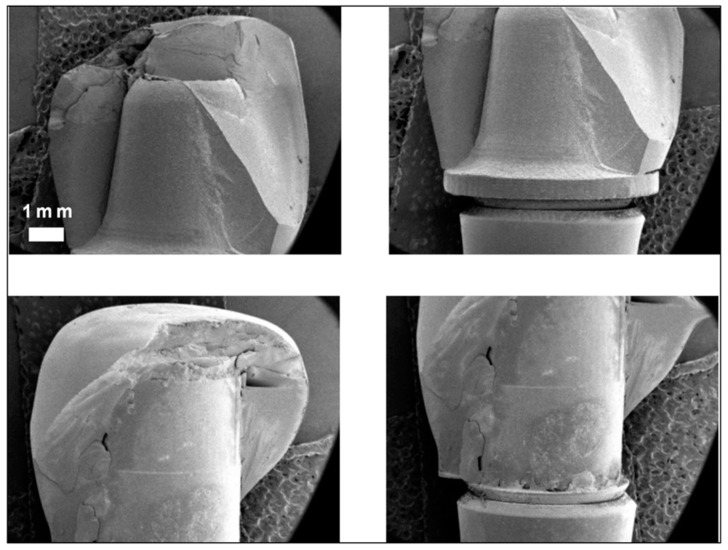
Fracture sections from sample group G2 (**upper** images) and G4 (**lower** images), respectively.

Figure 10 illustrates the fracture sections of fractured crowns pertaining to sample groups G2 and G4. A fractographic indicated a localized fracture initiation site at the crown’s uppermost region, presumably at the point of interaction with the load application clamp. Both samples exhibited crack formation on the external surface.

The fracture surface of sample G2 displayed superior material adherence. In contrast, the fracture surface of sample G4 exhibited interfacial cracks and areas with inadequate adherence, suggesting the existence of air bubbles between the material and the zirconia insert surface.

**Figure 11:**
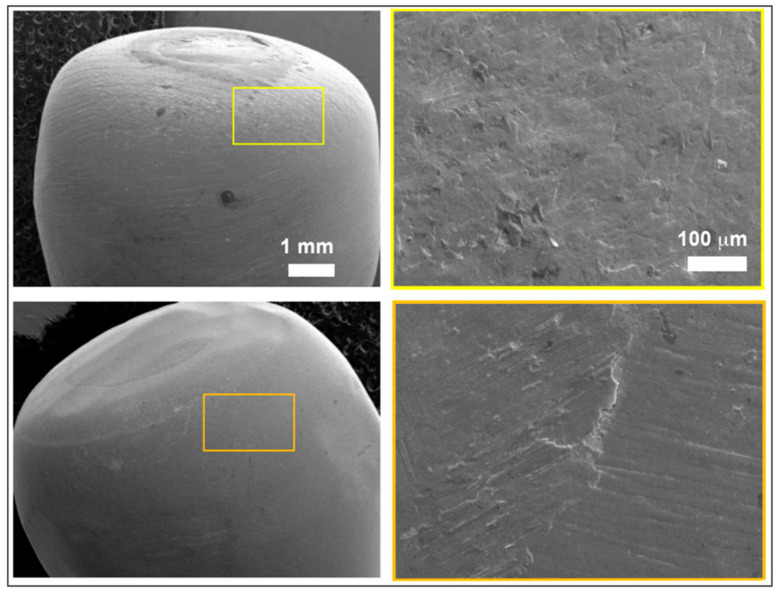
SEM images of unfractured samples of group G1 (**upper** images) and G3 (**lower** images).

Scanning Electron Microscopy (SEM) images of non-fractured crowns subjected to fatigue tests for sample groups G1 (images a and b) and G3 (images c and d) are presented in Figure 11, respectively. 

Detailed fractographic analysis of both specimens at higher magnifications (images b and d) shows a minimal contact area between the clamp and crown with no evidence of fissures, cracks, delamination or material detachment in the crown. 

The mechanical load applied to the top of the samples would have been efficiently transmitted to the crown/analog connection area without causing any adverse effects on the crown material beyond minor wear marks due to relative sliding between the clamp and crown at the contact point.

**Figure 12:**
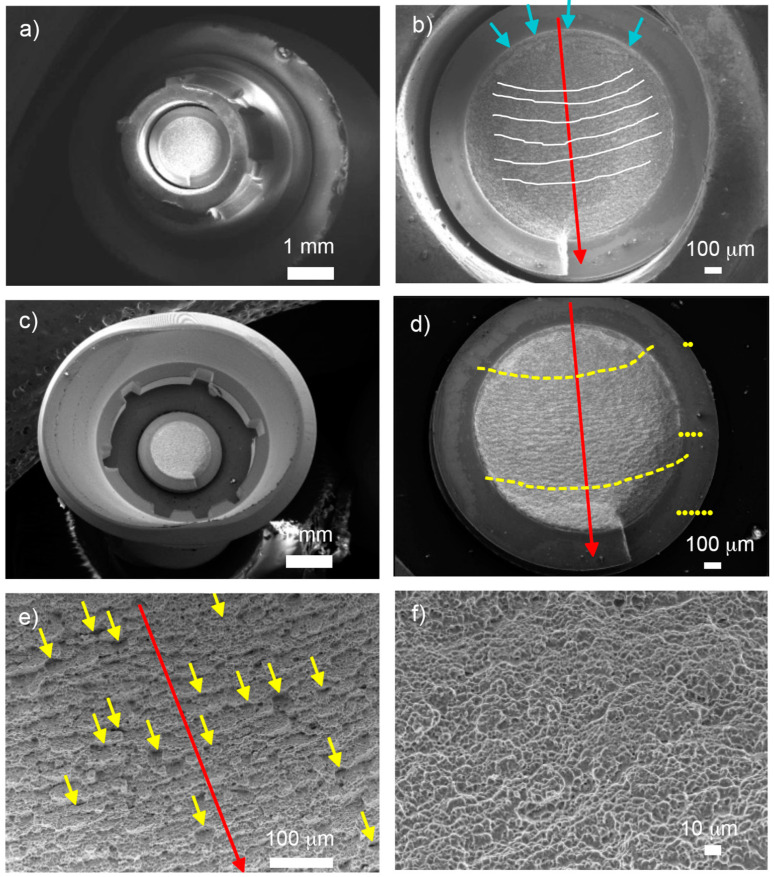
SEM images of fractured G1 samples, detailing: (**a**) abutment deformation, (**c**) fracture section of the screw with multiple crack initiation points, (**b**) analogous deformation, (**d**) detailed view of fracture regions within the screw’s fracture section, (**e**) secondary cracking and fatigue striations. Red arrows denote the direction of fracture propagation and (**f**) micro-cavity “dimples”.

As shown in Figure 12, there was a significant level of plastic deformation experienced in the crown/abutment connection area (a and c), which transitioned from a spherical geometry to a completely oval shape. The components tested exhibited fracture along the loading direction and underwent significant levels of deformation in the same direction. Three fracture regions were identified (d): Region I, the initial nucleation crack zone, above the uppermost yellow striped line; Region II, the stable fatigue crack growth zone, between the yellow striped lines; and Region III, the catastrophic overload fracture zone, below the lower yellow striped line.

The observation of Region II (d) revealed the presence of two characteristic aspects indicative of fatigue fracture processes: striations and secondary cracking. Striations appeared as cyclic fatigue loading patterns in the form of thin parallel lines (e), while secondary cracking manifested as arrays of parallel microcracks perpendicular to the main direction of fracture propagation. In the ultimate failure in Region III (f), the fracture became unstable, leading to an “overload fracture” characterized by significant plastic deformation and “dimple” micro-cavity formation on the fracture surface.

Figure 12 depicts the fractured sections of group G3, highlighting the same three fracture regions on the fracture surface (d), delineated by yellow dashed lines. Region I was located on the outer surface of the screw in the zone submitted to bending and tensile stresses. In addition, it exhibited a limited number of crack initiation points indicated by blue arrows (d). These initiation points were situated on the smooth outer surface of the screw near the body–head connection zone, specifically within the thread valley. Region II exhibited the largest fracture surface area among the three regions, in addition to the presence of typical fracture advancement beach marks: (c) indicated by white lines and typical secondary crack striations and (e) indicated by yellow arrows.

**Figure 13:**
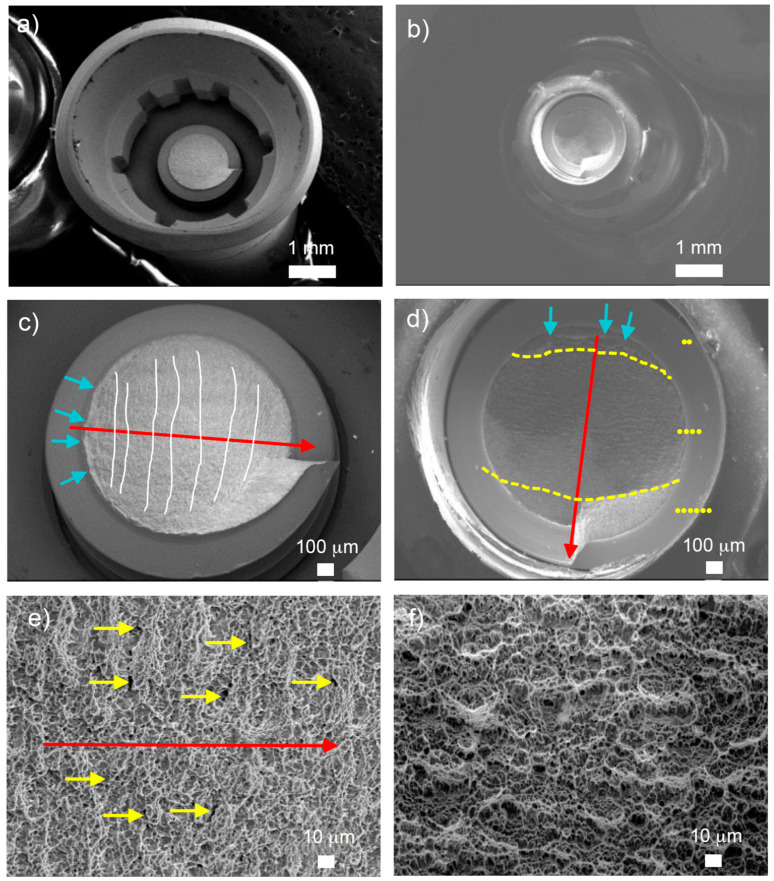
SEM images of fractured G3 samples. (**a**,**b**) Analogous and abutment deformation and screw’s fracture sections. (**c**) Multiple crack initiation points (blue arrows). (**d**) Detailed view of fracture regions within the screw’s fracture section. (**e**) Secondary cracking and fatigue striations Red arrows denote the direction of fracture propagation. (**f**) Micro-cavity “dimples”.

In Region II (Figure 13), beach marks were observed, and their positions are perpendicular to the path of fracture advancement, which helps locate the point of fracture initiation. The secondary cracks seemed to propagate in the same preferential direction perpendicular to both the fracture advancement direction and beach marks (e), gradually reducing the resistant section of the screw. Region III, situated in the lower section of the micrograph (d), displayed notable plastic deformation from mechanical overloading, leading to the formation of “dimple” micro-cavities (f). This rougher region featured granular morphology and numerous dimples characteristic of microvoid coalescence.

**Figure 14:**
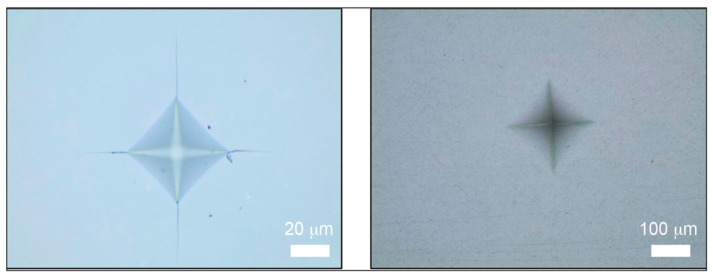
Images of indentations captured through optical microscopy. (**Right**) Low-magnification imprint obtained in a composite region of a dental abutment pillar. (**Left**) High-magnification imprint obtained in a zirconia region of a dental abutment pillar.

The indentation tests on the zirconia regions resulted in “small” imprints with a diameter of approximately 82 µm, while the same tests on the composite regions produced much larger imprints with a diameter of 355 µm (Figure 14). Contrarily, no cracks were observed in the composite regions at the corners of the imprints that prevented the determination of fracture toughness in these areas.

As shown in Table 6, the zirconia regions exhibited the standard hardness and fracture toughness values for dental zirconia ceramic grades, while the composite regions displayed lower hardness values.

## 4. Discussion

The main objective was to evaluate the mechanical behavior of the different combinations of materials among the samples and see how it affects the failure mode under static and dynamic loading.

Since the beginning of dental implantology history, the lack of cushioning on the ankylosis of implants has been a great concern and represents a big challenge for industrial manufacturers. The first attempt was the IMZ implant with the intra-mobile element (IME) as its implant system, which was very popular in the 1980s [56]; another different prototype was presented in 2014 [57]. New developments in CAD/CAM materials, especially resin-based blocks, appeared on the market in the same decade. In 2012, the global manufacture of dental materials (3M ESPE, Seefeld, Germany) launched a new CAD/CAM restorative material: Lava Ultimate, based on Magne et al. [24] which was the first study that combined stiff ceramic and the resilient composite block. Despite the promising results of this combination of materials in emulating the Cushing effect of the periodontal ligament, the results from one clinical trial presented a higher failure rate, approximately 80% [58]. Two in vitro studies by Krejci et al. [59] and Lohbauer et al. [60] explained the reasons for the failures by demonstrating the high stress concentration at the bonding interface in between composite/zirconia and the reasons for debonding.

Thus, combining resilient composite on top of the implant crown with a low module of elasticity (around 10–15 Gpa of resin-composite blocks) and stiff ceramic with a high module of elasticity (210 Gpa of zirconia) on the base of the implant crown and bonded to the Ti-base abutment needs adequate bonding to work accordingly with the materials. Hence, in 2015, the same manufacturer withdrew the indication for implant and dental crowns, given the lack of a bonding strategy. Later, in 2016, a new resin block was launched in the dental market along with a new bonding strategy: Brilliant Crios and One Coat 7 Universal (Coltene Whaledent, Altstätten, Switzerland). The effectiveness of the new bonding protocol was confirmed by Reymus et al. [61] and Emsermann et al. [62], showing the disadvantages of using silane over resin-composite blocks and the benefits of monomers containing MDP.

Currently, the use of resin-composite blocks for implant crowns is well accepted because of the damping effect, considering the high occlusal forces of around 900 N required for molar areas [63,64,65]. Given the lack of evidence, it is unknown which is the best scenario for this material: either the monolithic bonded directly to the Ti-base (G3) or combined with mesostructured zirconia (G2 and G4), as proposed by Magne et al. [24]. According to our results, both scenarios—monolithic G2 or layered G3 and G4—demonstrated fracture strength in comparison to zirconia monolithic ceramic crowns G1, with no statistically significant differences in Fmax.

The key point of this study was focused on the damping effect and the failure mode of the material combination crown–abutment. This influenced the failure mode of the restoration, transitioning from a fatigue fracture type located at the abutment–analog connection for monolithic materials (G1 and G3) to a brittle fracture located in the crown for bi-layered materials (G2 and G4). This coincides with the research carried out by Elsayed et al. [35], where they demonstrated favorable failures, and Taha et al. [34], who concluded that with less rigid crown materials, a stiff substructure might be able to preserve their force absorption behavior.

This study was conducted to simulate the chewing function using cyclic loading and the humidity of the oral cavity conditions to ensure their reliability and durability. We also used prefabricated blocks and original Ti-base abutments with a conical abutment connection [66,67,68] to achieve the ideal tolerance, cement space and a proper bonding strategy between the Ti-base and the different layers of the implant crowns under study. Thus, to achieve the damping effect in resin implant restorations with a low module of elasticity, it is necessary to have support from a stiff material, such as a substructure or mesostructure, in contact with the titanium base, as demonstrated by Südbeck et al. [69]. Furthermore, a reliable bonding interface among the different materials is essential, as confirmed by Rosentritt et al. [23].

The main hypothesis of this study was validated twice through the experimental findings, as significant differences had been observed among the four assessed materials within implant–abutment–screw assemblies, both in terms of maximum fracture strength and fatigue limit values. Consequently, the null hypothesis was rejected due to the discernment of significant differences among the sample groups, as proved by the results of analyses of fracture resistance, fatigue survival, and fracture mode. This makes them potentially suitable as an alternative for restoring single implants, even in the posterior area of the mouth. The fatigue limits of the four tested groups have been determined, with the resistance arranged in the following decreasing order: G1 > G4 > G2 > G3.

Future research works are expected to focus on increasing the fracture and fatigue resistance of these bi-layered crowns through a geometric overhaul of the internal ceramic insert. This dual objective aims to amplify load-absorption capacity and refine internal load distribution, thereby concurrently reducing potential stress concentration effects.

The main limitations of the study are, among others, the use of implant replicas instead of real implants, the use of implant crowns with bigger prosthetic height and testing the wear of the implant composite block. Those aspects should be laboratory and clinically tested to evaluate the long-term performance of these restorations in the future scope of current work.

## 5. Conclusions

From the results obtained from both static and dynamic mechanical tests, the use of monolithic crowns would entail the direct and complete transmission of applied mechanical loads to the crown/abutment connection area, leading to progressive deformation of the abutment neck and eventual fatigue fracture of the connection screw. On the other hand, the use of bi-laminar crowns appears to protect the crown/abutment connection area by partially absorbing the applied mechanical loads, preventing deformation and fracture of the connection area at the expense of facing the final partial or total fracture of the crown.

The maximum fracture strength values obtained in this study greatly surpass the previously reported maximum beat occlusal forces, with mean Fmax values ranging from 1510.20 ± 176.96 N to 1671.18 ± 119.17 N, corresponding to the sample groups G2 and G3, respectively. Additionally, in a comparative context, the fatigue limit (LF) values obtained in this study, ranging between 668 N and 813 N, would be of a comparable magnitude, potentially indicative of infinite fatigue life resistance without failure.

Both monolithic and bi-laminar designs are approved for dental crown use, even in high-stress molar regions. Bi-laminar crowns, however, seem to safeguard the crown/abutment junction by absorbing mechanical loads, averting excessive deformation and implant fracture. This enables the crown repair of post-partial or full fractures without the need for implant removal. 

In future studies, efforts are expected to improve the fracture and fatigue resistance of these bi-laminar crowns by geometrically redesigning the inner ceramic insert to enhance load distribution and reduce potential stress concentration.

## 6. Patents

The authors, Dr. Nicolas Gutierrez R. and Dr. Ralf Böhner (RIP), declare the rights as inventors of the “DENTAL BLANK WITH AN INSERT” by the European patent # 17175940.0-1126 on 19 September 2018 and US patent # 11.633,267 B2 in 25 April 2023.

## Figures and Tables

**Table 1:** General table of the material properties for each study sample group.

Materials	Manufacturers	Composition	Flexural Strength	Fracture Toughness	Modulus of Elasticity
Zirconia crown: CEREC Zirconia meso L A2 LOT 2017484418	Dentsply Sirona®	Yttria stabilized zirconia	>900 MPa	7.1 MPa^1/2^	210 GPa
Composite crown: BRILLIANT Crios Disc A2 LT H18 LOT J52869	Coltene®	Barium glass < 1.0 µm, amorphous silica SiO_2_ < 20 nm, cross linked methacrylic matrix and inorganic pigments	198 MPa	2.0 MPa m0.5	10.3 GPa
Customized meso structure: inCoris ZI meso F2 L LOT 3314000426	Dentsply Sirona®	Dentsply Sirona®	>900 MPa	5.8 MPa^1/2^	210 GPa
Zirconia insert	Coltene®	Yttria stabilized zirconia (3 mol% Y2O3)	500–1000 MPa	5.8–10.5 MPa m0.5	210 GPa
TiBase CEREC/inLab AT EV 4,8 GH 1 “L” LOT B200003054	Dentsply Sirona®	Ti6Al4V, medical grade 5, ASTM 136	n.a	n.a	105–117 GPa
Implant Replica EV 5.4 LOT 456009	Dentsply Sirona®	Ti6Al4V Grade 5 ASTM F136	n.a	n.a	105–117 GPa
Cement: Solocem® LOT J64901	Coltene®	UDMA, TEGDMA, HEMA Methacrylate, zinc oxide, dental glass, MDP and 4-MET (A) monomers	120 MPa	n.a	7.2 GPa
Bonding: OneCoat 7 Universal® LOT J69945	Coltene®	Methacrylates including 10-MDP photoinitiators, ethanol, water	n.a	n.a	n.a

**Table 2:** Descriptive table of the surface treatments used for sample adhesion.

Surface Pre-Treatment Group	G1	G2	G3	G4
**Ti-base**	Sand-blasting 2.5 bar, Al_2_O_3_, particles, 50 µm			
**Monolithic zirconia crown**	Sand-blasting, 1 bar, Al_2_O_3_ particles, 50 µm,			
**Composite crown**		Sand-blasting, 2 bar, Al_2_O_3_ particles, 50 µm,		
**Zirconia mesostructure**		Sand-blasting, 1 bar, Al_2_O_3_ particles, 50 µm,		
**Zirconia insert**				Sand-blasting, 1 bar, Al_2_O_3_ particles, 50 µm,
**Primer/Cement**	OneCoat 7 universal and Solocem by Coltene^®^			

**Table 3:** General table of maximum compressive force and elongation at break values determined by static compression.

Properties/Group of Samples	N°	G1	G1	G3	G4
**Fmax, N**	1	1706.10	1699.50	1720.10	1572.20
	2	1578.60	1397.70	1717.10	1701.40
	3	1737.10	1439.70	1818.10	1487.40
	4	1565.00	1696.50	1523.00	1549.60
	5	1546.10	1317.60	1577.60	1457.10
	$\bar{X}$	1626.58	1510.20	1671.18	1553.54
	D. Std	88.19	176.96	119.17	94.74
**Elongation at break, mm**	1	1.25	0.95	1.32	0.67
	2	1.29	0.60	1.23	0.88
	3	1.17	1.06	1.28	1.16
	4	1.26	1.40	1.50	1.07
	5	1.31	0.94	1.35	0.80
	$\bar{X}$	1.26	0.99	1.34	0.92
	D. Std	0.05	0.29	0.10	0.20

**Table 4:** Comparative table of fatigue limit values (FL) obtained for all groups of samples tested.

Property/Group of Samples	G1	G2	G3	G4
**FL, N**	813	755	668	777

**Table 5:** Maximum and minimum loads supported by each group sample illustrating up to 8 distinct fracture modes.

Group of Samples	% Fmax	Fmax (N)	Fmin (N)	N° Cycles to Break	Failure Mode
G1	80	1301	130	16,314	T2
	80	1301	130	30,475	T4
	70	1139	114	105,522	T6
	70	1139	114	75,315	T5
	60	976	98	470,741	T7
	60	976	98	805,596	T8
	50	813	81	2,000,000	Run out
	50	813	81	2,000,000	Run out
G2	80	1208	121	16,028	T5
	80	1208	121	16,930	T5
	70	1057	106	109,106	T5
	70	1057	106	148,110	T6
	60	906	91	1,631,627	T5
	60	906	91	273,531	T6
	50	755	76	2,000,000	Run out
	50	755	76	2,000,000	Run out
G3	80	1337	134	3797	T5
	80	1337	134	9108	T6
	60	1003	100	43,309	T5
	60	1003	100	140,250	T6
	50	836	84	1,197,943	T5
	50	836	84	1,864,478	T6
	40	668	67	2,000,000	Run out
	40	668	67	2,000,000	Run out
G4	80	1243	124	1724	T1
	80	1243	124	1396	T5
	70	1087	109	2063	T3
	70	1087	109	4518	T2
	60	932	93	265,460	T1
	60	932	93	328,837	T4
	50	777	78	2,000,000	Run out
	50	777	78	2,000,000	Run out

Where: T1: (Partial fracture of the crown), T2: (Partial fracture of the crown and fracture of screw, with deformation of both implant and abutment), T3: (Total fracture of the crown, without deformation of either the implant or the abutment), T4: (Total fracture of the crown, with deformation of both implant and abutment), T5: (Fracture of the screw, with deformation of both implant and abutment), T6: (Fracture of the screw, with deformation of the abutment and partial fracture of the implant), T7: (Fracture of the screw, with deformation of the abutment and total fracture of the implant).

**Table 6:** Summary of HV5 hardness and KIC fracture toughness characterization.

Sample Groups	Region	HV5 (GPa)	*K_IC_* (MPa·m^1/2^)
G1	Zirconia	13.7 ± 0.3	5.0 ± 0.3
G2	Zirconia (internal region)	13.6 ± 0.2	4.6 ± 0.4
	Composite (external region)	0.7 ± 0.1	-
G3	Composite	0.7 ± 0.1	-
G4	Zirconia (internal region)	13.8 ± 0.3	4.6 ± 0.4
	Composite (external region)	0.7 ± 0.1	-

## Data Availability

The original contributions presented in the study are included in the article, further inquiries can be directed to the corresponding author.
